# Performance and Sensitivity of [^99m^Tc]Tc-sestamibi Compared with Positron Emission Tomography Radiotracers to Measure P-glycoprotein Function in the Kidneys and Liver

**DOI:** 10.1021/acs.molpharmaceut.3c01036

**Published:** 2024-01-16

**Authors:** Irene Hernández-Lozano, Sarah Leterrier, Severin Mairinger, Johann Stanek, Anna S. Zacher, Lara Breyer, Marcus Hacker, Markus Zeitlinger, Jens Pahnke, Nicolas Tournier, Thomas Wanek, Oliver Langer

**Affiliations:** † Department of Clinical Pharmacology, Medical University of Vienna, 1090 Vienna, Austria; ‡ Laboratoire d’Imagerie Biomédicale Multimodale (BIOMAPS), Université Paris-Saclay, CEA, CNRS, Inserm, Service Hospitalier Frédéric Joliot, 91401 Orsay, France; § Department of Biomedical Imaging and Image-guided Therapy, Medical University of Vienna, 1090 Vienna, Austria; ∥ Department of Pathology, Section of Neuropathology, Translational Neurodegeneration Research and Neuropathology Lab, University of Oslo (UiO) and Oslo University Hospital (OUS), 0372 Oslo, Norway; ⊥ Lübeck Institute of Experimental Dermatology (LIED), Pahnke Lab, University of Lübeck and University Medical Center Schleswig-Holstein, 23538 Lübeck, Germany; # Department of Pharmacology, Faculty of Medicine, University of Latvia, 1004 Ri̅ga, Latvia; ∇ Department of Neurobiology, The Georg S. Wise Faculty of Life Sciences, Tel Aviv University, 6997801 Tel Aviv, Israel

**Keywords:** PET, SPECT, P-glycoprotein, drug–drug interaction, probe substrate, [99 mTc]Tc-sestamibi

## Abstract

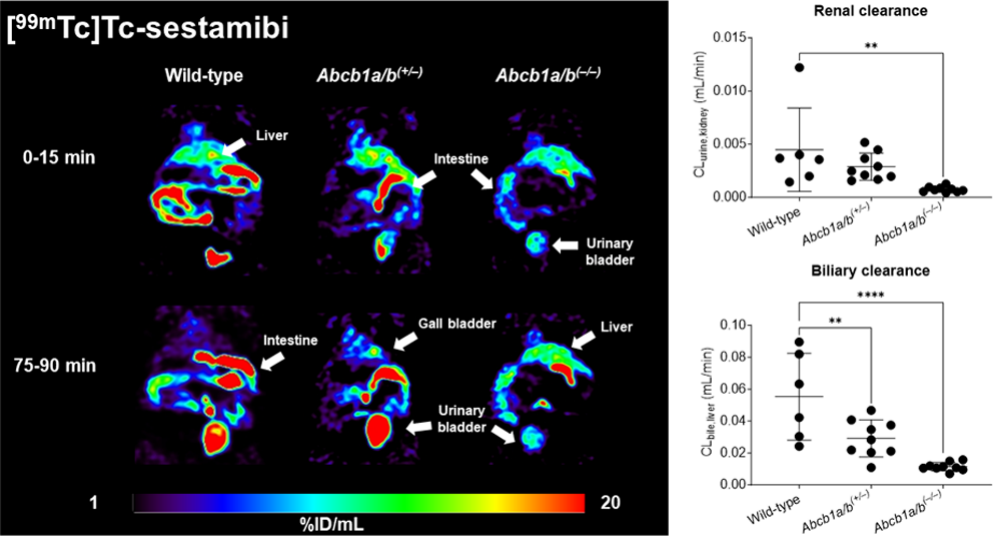

P-glycoprotein (P-gp, encoded in humans by the *ABCB1* gene and in rodents by the *Abcb1a/b* genes) is a membrane transporter that can restrict the intestinal absorption and tissue distribution of many drugs and may also contribute to renal and hepatobiliary drug excretion. The aim of this study was to compare the performance and sensitivity of currently available radiolabeled P-gp substrates for positron emission tomography (PET) with the single-photon emission computed tomography (SPECT) radiotracer [^99m^Tc]Tc-sestamibi for measuring the P-gp function in the kidneys and liver. Wild-type, heterozygous (*Abcb1a/b*^*(+/***–***)*^), and homozygous (*Abcb1a/b*^*(***–***/***–***)*^) *Abcb1a/b* knockout mice were used as models of different P-gp abundance in excretory organs. Animals underwent either dynamic PET scans after intravenous injection of [^11^C]*N*-desmethyl-loperamide, (*R*)-[^11^C]verapamil, or [^11^C]metoclopramide or consecutive static SPECT scans after intravenous injection of [^99m^Tc]Tc-sestamibi. P-gp in the kidneys and liver of the mouse models was analyzed with immunofluorescence labeling and Western blotting. In the kidneys, *Abcb1a/b*^*()*^ mice had intermediate P-gp abundance compared with wild-type and *Abcb1a/b*^*(−/−)*^ mice. Among the four tested radiotracers, renal clearance of radioactivity (CL_urine,kidney_) was significantly reduced (−83%) in *Abcb1a/b*^*(***–***/***–***)*^ mice only for [^99m^Tc]Tc-sestamibi. Biliary clearance of radioactivity (CL_bile,liver_) was significantly reduced in *Abcb1a/b*^*(***–***/***–***)*^ mice for [^11^C]*N*-desmethyl-loperamide (−47%), [^11^C]metoclopramide (−25%), and [^99m^Tc]Tc-sestamibi (−79%). However, in *Abcb1a/b*^*(+/***–***)*^ mice, CL_bile,liver_ was significantly reduced (−47%) only for [^99m^Tc]Tc-sestamibi. Among the tested radiotracers, [^99m^Tc]Tc-sestamibi performed best in measuring the P-gp function in the kidneys and liver. Owing to its widespread clinical availability, [^99m^Tc]Tc-sestamibi represents a promising probe substrate to assess systemic P-gp-mediated drug–drug interactions and to measure renal and hepatic P-gp function under different (patho-)physiological conditions.

## Introduction

P-glycoprotein (P-gp, encoded in humans by the *ABCB1* gene and in rodents by the *Abcb1a/b* genes) is an ATP-binding cassette (ABC) transporter that is abundantly expressed in excretory organs (i.e., the kidneys and liver), in the intestine, and at blood–tissue barriers, such as the blood–brain barrier (BBB).^1^ P-gp recognizes many structurally unrelated small-molecule drugs and their metabolites as substrates. P-gp can limit the intestinal absorption of orally administered drugs, restrict the brain distribution of certain drugs at the BBB, and may contribute to the renal and hepatobiliary excretion of drugs.

Given its important role in drug disposition, P-gp can be involved in drug–drug interactions (DDIs), in which the coadministration of a P-gp substrate (victim) drug and a P-gp inhibiting (perpetrator) drug may lead to altered disposition of the victim drug.^1^ As this may pose a considerable safety risk, it has become mandatory to investigate the risk for P-gp-mediated DDIs in drug development.^1^ The role of P-gp in intestinal DDIs leading to increased systemic absorption of certain substrate drugs is well established.^2^ On the other hand, whether P-gp-mediated DDIs can affect the renal and hepatobiliary excretion of drugs is less well understood.^3^

Whereas the consequences of intestinal P-gp-mediated DDIs on drug disposition can be straightforwardly assessed by measuring the plasma pharmacokinetics of the victim drugs, the analysis of systemic drug exposure alone may not be suitable to directly assess the impact of renal and hepatic P-gp-mediated DDIs on drug disposition. Urine can be collected to estimate the importance of P-gp in mediating the urinary clearance of drugs in humans.^4^ However, the total collection of bile is hardly achievable in humans to assess the contribution of P-gp to the biliary excretion of its substrates. As an alternative approach, the nuclear medicine imaging techniques positron emission tomography (PET) and single-photon emission computed tomography (SPECT) have been proposed, which enable the dynamic measurement of the tissue concentrations of radiolabeled drugs.^5,6^

A few radiolabeled P-gp substrates (i.e., [^11^C]*N*-desmethyl-loperamide, racemic or (*R*)-[^11^C]verapamil, [^11^C]metoclopramide, and [^18^F]MC225) have been used to assess P-gp-mediated DDIs at the human BBB with PET imaging, employing cyclosporine A, tariquidar, or quinidine as P-gp inhibitors.^7−12^ In contrast to the brain, PET imaging has so far not been validated to assess P-gp-mediated DDIs in the kidneys and the liver. On the other hand, the myocardial perfusion SPECT radiotracer [^99m^Tc]Tc-sestamibi, which is a substrate of P-gp,^13^ has been used to assess P-gp-mediated DDIs in the human liver.^14−16^ A radiolabeled P-gp probe substrate that allows for an imaging-based measurement of P-gp function in the kidneys and liver may be of use for a detailed mechanistic assessment of the *in vivo* DDI perpetrator risk of drug candidates that inhibit P-gp *in vitro*. Moreover, such a radiolabeled P-gp probe substrate could be used to assess the influence of intrinsic factors, such as disease or genetic polymorphisms, on renal and hepatic P-gp function.

The aim of this study was to compare the performance and sensitivity of three radiolabeled P-gp substrates for PET (i.e., [^11^C]*N*-desmethyl-loperamide, (*R*)-[^11^C]verapamil, and [^11^C]metoclopramide) with [^99m^Tc]Tc-sestamibi for measuring P-gp function in the kidneys and liver of mice. Since a complete loss of P-gp, as it occurs in *Abcb1a/b*^*(−/−)*^ mice, represents a drastic scenario that does not reflect a clinically realistic reduction in P-gp function caused by DDIs or disease, we included heterozygous *Abcb1a/b* knockout (*Abcb1a/b*^*()*^) mice into our evaluation as a model of moderately reduced P-gp function.^17^

## Experimental Section

### Chemicals and Radiotracer Synthesis

Unless otherwise stated, chemicals were purchased from Sigma-Aldrich or Merck. Metoclopramide ampules (Paspertin, 10 mg/2 mL) were obtained from a local pharmacy. [^11^C]*N*-desmethyl-loperamide, (*R*)-[^11^C]verapamil, and [^11^C]metoclopramide were synthesized as previously described.^18−20^ For intravenous (i.v.) injection into mice, [^11^C]*N*-desmethyl-loperamide and (*R*)-[^11^C]verapamil were formulated in physiological saline solution (0.9%, w/v) containing 0.1% (v/v) polysorbate 80 and [^11^C]metoclopramide was formulated in physiological saline solution containing 0.05 mg of unlabeled metoclopramide (Paspertin) to slow down the metabolism of [^11^C]metoclopramide.^21^ [^99m^Tc]Tc-sestamibi was prepared by labeling of Medi-MIBI 500 Mikrogramm kits (Radiopharmacy Laboratory Ltd., Budaörs, Hungary) with [^99m^Tc]pertechnetate according to the manufacturer’s instructions and formulated in physiological saline solution for i.v. injection to mice.

### Animals

Female wild-type *Abcb1a/b*^*(*+/***–**)*^ and *Abcb1a/b*^*(−/−)*^ mice, all with a C57BL/6J genetic background, were generated at the KPM Radium Hospital (Oslo, Norway). Animals were housed in type III IVC cages under controlled environmental conditions (22 ± 3 °C, 40–70% humidity, 12 h light/dark cycle) and had free access to standard laboratory rodent diet and water. An acclimatization period of at least 1 week was allowed before the animals were used in the experiments. The animal experiments were either approved by the Intramural Committee for Animal Experimentation of the Medical University of Vienna and the Austrian Federal Ministry of Education, Science and Research [2021-0.785.873] or by the Amt der Niederösterreichischen Landesregierung. All study procedures were performed in accordance with the European Community’s Council Directive of 22 September 2010 (2010/63/EU).

### PET Imaging

The PET data sets analyzed in this study were previously published by Wanek et al.,^22^ Zoufal et al.,^23^ and Mairinger et al.^17^ In these previous studies, only the brain distribution of the PET radiotracers was analyzed in different mouse models. An overview of the mouse groups included in this study is given in Table 1.

**Table 1:** Overview of the Mouse Groups Included in the Study^a^

		*n* included	weight (g)	injected activity (MBq)
[^11^C]*N*-desmethyl-loperamide	wild-type	6	21.6 ± 1.5	36.0 ± 5.3
	*Abcb1a/b*^*(****+****/***–***)*^	3	23.9 ± 1.1	43.9 ± 3.6
	*Abcb1a/b*^*(***–***/***–***)*^	4	20.4 ± 0.6	31.7 ± 8.0
(*R*)-[^11^C]verapamil	wild-type	5	22.6 ± 1.2	36.3 ± 2.1
	*Abcb1a/b*^*(****+****/***–***)*^	4	23.4 ± 2.0	35.3 ± 6.0
	*Abcb1a/b*^*(***–***/***–***)*^	4	21.8 ± 0.6	34.2 ± 3.3
[^11^C]metoclopramide	wild-type	6	23.0 ± 1.4	34.5 ± 2.1
	*Abcb1a/b*^*(****+****/***–***)*^	6	21.0 ± 0.7	38.7 ± 9.7
	*Abcb1a/b*^*(***–***/***–***)*^	6	21.2 ± 1.1	36.5 ± 11.5
[^99m^Tc]Tc-sestamibi	wild-type	6	29.9 ± 3.3	35.5 ± 9.5
	*Abcb1a/b*^*(****+****/***–***)*^	9	31.6 ± 5.3	38.9 ± 4.0
	*Abcb1a/b*^*(***–***/***–***)*^	8	27.5 ± 3.1	40.2 ± 8.9

a Continuous data are given as mean ± standard deviation (SD).

Wild-type, *Abcb1a/b*^*(**+**/***–***)*^, and *Abcb1a/b*^*(***–***/***–***)*^ mice underwent under isoflurane/air anesthesia 60 min dynamic PET scans after i.v. administration of [^11^C]*N*-desmethyl-loperamide, (*R*)-[^11^C]verapamil, or [^11^C]metoclopramide using a microPET Focus 220 scanner (Siemens Medical Solutions) as previously described.^17,22,23^ At the end of the PET scan, a blood sample was collected from the retro-bulbar plexus, and animals were killed by cervical dislocation while still under deep anesthesia. Radioactivity in weighted blood aliquots was measured on a gamma counter. From two animals per genotype, the kidneys and the liver were removed and snap-frozen in liquid nitrogen-cooled isopentane and stored at −80 °C for immunofluorescence labeling and Western blot analysis of P-gp.

### SPECT/CT Imaging

SPECT/computed tomography (CT) scans using a mouse whole-body pinhole collimator (5 × 1.0 mm, transaxial field of view: 6 cm) were performed on an Inveon multimodality microPET/SPECT/CT system (Siemens Medical Solutions USA, Inc.). Wild-type, *Abcb1a/b*^*(**+**/***–***)*^, and *Abcb1a/b*^*(***–***/***–***)*^ mice (Table 1) underwent under isoflurane anesthesia (2.5–3.5% (v/v) in medical air) after i.v. administration of [^99m^Tc]Tc-sestamibi six consecutive 15 min static SPECT scans followed by a CT scan for attenuation correction and anatomical visualization (total acquisition time: approximately 105 min). As animals were inaccessible when positioned in the gantry of the scanner, i.v. [^99m^Tc]Tc-sestamibi injections were performed while the bed was outside the scanner followed by moving the bed into the scanner and the start of the SPECT acquisition. The mean time delay between the radiotracer injection and the start of the first static SPECT scan was 8 ± 4 min (range: 4–18 min). List mode data were acquired with an energy window of 126–154 keV. During the entire experiment, animals were warmed and their body temperature and respiratory rate were constantly monitored. Isoflurane concentration was varied during image acquisition to maintain the animals at a constant breathing rate of 60–80 breaths per minute. At the end of the CT scan, a blood sample was collected from the retro-bulbar plexus and animals were killed by cervical dislocation while still under deep anesthesia. Radioactivity in weighted blood aliquots was measured in a gamma counter.

#### Analysis of Imaging Data

Dynamic PET data were sorted into 23 time frames with a duration of 5 s to 10 min. PET images were reconstructed using Fourier rebinning of the three-dimensional sinograms followed by a two-dimensional filtered back-projection with a ramp filter giving a voxel size of 0.4 × 0.4 × 0.796 mm^3^. SPECT images were reconstructed using a three-dimensional *maximum a posteriori* (MAP3D) algorithm with 6 subsets/16 iterations resulting in a final voxel size of 0.5 × 0.5 × 0.5 mm^3^. The standard data correction protocol, including normalization, attenuation and decay correction, was applied to the data. SPECT data were additionally scatter-corrected by using the SPECT Triple Energy Window (TEW) method. Images were analyzed with the medical image data examiner software AMIDE.^24^ Liver, left kidney, gall bladder, intestine (representing all of the visible intestinal radioactivity), and urinary bladder (assumed to represent excreted urine) were manually outlined on the reconstructed and coregistered (SPECT/CT) images [see the Supporting Information (SI), Figure S1]. Time–activity curves were extracted for each outlined region of interest (ROI) and expressed as percent of the injected dose per mL (%ID/mL) for the kidney and liver and as percent of the injected dose (%ID) for the urinary bladder and intestine, by multiplication of the image-derived radioactivity concentration with the ROI volume. Since the static [^99m^Tc]Tc-sestamibi SPECT scans were not acquired at exactly the same time points after radiotracer injection in different animals, time–activity curves were interpolated in all animals to the same time points using Microsoft Excel 2019 (version 2311). It was assumed that the sum of radioactivity in the gall bladder and the intestine represented radioactivity in the bile excreted from the liver and that the direct secretion of radioactivity from blood into the intestine was negligible over the short duration of the imaging experiments. To confirm this assumption, it would be necessary to examine bile-duct-cannulated mice, which is technically challenging and was not performed in the present study.

### Kinetic Analysis

The area under the time–activity curve (AUC, %ID/mL × min) was calculated using Prism (version 8.0, GraphPad). The renal and biliary clearances (CL_urine,kidney_ and CL_bile,liver_, mL/min) with respect to the kidney and the liver concentrations, respectively, were calculated by dividing the total amount of radioactivity at the last imaging time point (i.e., 60 min for PET and 98 min for SPECT) in the urinary bladder or intestine by AUC_kidney_ or AUC_liver_, respectively.^25^

### Immunofluorescence Labeling

Frozen kidney and liver tissue were cut into 14 μm thick slices and mounted on slides. The slides were incubated for 15 min at room temperature (rt) in 4% (w/v) aqueous paraformaldehyde (PFA). To block the PFA, the slides were incubated for 5 min at rt in phosphate-buffered saline (PBS) containing 50 mM of ammonium chloride. Then the cuts were permeabilized with frozen methanol/acetone (1/1, 5 min, −20 °C, Carlo Erba Reagents, France) followed by PBS solution containing 0.1% (v/v) Triton X-100 (5 min, rt). Several washes with PBS were carried out between each of these steps. The nonspecific sites were saturated by incubating the slides for 1 h at rt in a PBS solution containing 5% (v/v) bovine albumin serum and 0.5% (v/v) Tween 80. Each slide was incubated for 1 h in the presence of an anti-P-gp primary antibody (1:100, anti-P-glycoprotein recombinant rabbit monoclonal antibody, clone ARC0470, #MA5-35257, Thermo Fisher Scientific). After several washes with PBS to remove excess antibody, the slides were left for 30 min at rt in the presence of a solution containing the secondary antibody (1:1000, goat antirabbit AlexaFluor 488, #Ab150077, Thermo Fisher Scientific). Finally, all of the slides were rinsed again with PBS, and then mounted with a mounting medium containing 4′,6-diamidino-2-phenylindole (DAPI) for labeling the nuclei (ProLong Diamond Antifade Mountant with DAPI, Thermo Fisher Scientific). Kidney and liver sections were scanned with a 20× objective using an AxiObserver Z1 microscope (Carl Zeiss AG, Germany). Intensity measurement of the fluorescent signal was performed with ImageJ 1.53s software (https://imagej.nih.gov/ij/). In each section, the whole fluorescence intensity was corrected for background.

### Western Blot

Kidney or liver tissue was homogenized in cell lysis buffer (#9803, Cell Signaling Technology) containing proteases/phosphatases inhibitor cocktail (Halt Protease and Phosphatase Inhibitor Cocktail (100×), Thermo Fischer). Samples were centrifuged at 14,000*g* for 15 min at 4 °C. Protein concentrations were determined using the Bradford protein assay (Thermo Fisher). Western blots were performed using 4–20% Mini-protein TGX Precast Protein Gels and the Trans-blot turbo transfer system (Bio-Rad Laboratories). After protein transfer, unspecific binding sites were blocked by incubation in Tris-buffered saline with Tween 20 (TBS-Tween; 50 mM Tris/HCl, 150 mM NaCl, 0.05% (w/v) Tween 20) containing 5% skimmed milk for 1 h at rt. Blotting membranes were incubated overnight at 4 °C with anti-P-gp primary antibody (1:100, clone ARC0470, #MA5-35257, Thermo Fisher Scientific) and α-tubulin antibody (1:1,000, #2144, Cell Signaling Technology) in 5% skimmed milk TBS-Tween. Then blotting membranes were incubated for 1 h with horseradish peroxidase (HRP)-conjugated secondary antirabbit antibody (1:10,000, #111-035-144, Jackson ImmunoResearch Laboratories, in 5% skimmed milk TBS-Tween). The target protein was revealed by the chemiluminescent HRP substrate (Clarity Western ECL Substrate, #1705060, Bio-Rad Laboratories). None of the Western blots shown were modified by nonlinear adjustments. Quantification was performed on scanned immunoblots using ImageJ 1.53s software. P-gp intensity values were normalized to α-tubulin intensity values and expressed as a percent of control (i.e., wild-type animals). For each mouse model, two animals were analyzed with two technical replicates each.

### Statistical Analysis

Statistical analysis was performed using Prism (version 8.0). The normal distribution of the values was assessed by visual inspection and the Shapiro–Wilk test. Differences in pharmacokinetic parameters between mouse groups were assessed by one-way analysis of variance (ANOVA) followed by a Dunnett’s multiple comparison test against the wild-type group. The level of statistical significance was set to a *p*-value of less than 0.05. All values are given as the mean ± standard deviation (SD).

## Results

We performed 60 min dynamic PET scans after i.v. administration of [^11^C]*N*-desmethyl-loperamide, (*R*)-[^11^C]verapamil, or [^11^C]metoclopramide or 6 consecutive 15 min static SPECT scans after i.v. administration of [^99m^Tc]Tc-sestamibi in wild-type, *Abcb1a/b*^*(+/***–***)*^, and *Abcb1a/b*^*(***–***/***–***)*^ mice. At the end of the [^11^C]metoclopramide PET scans, the kidneys and livers were collected from the three mouse models, and P-gp abundance was analyzed with immunofluorescence labeling and Western blotting.

**Figure 1:**
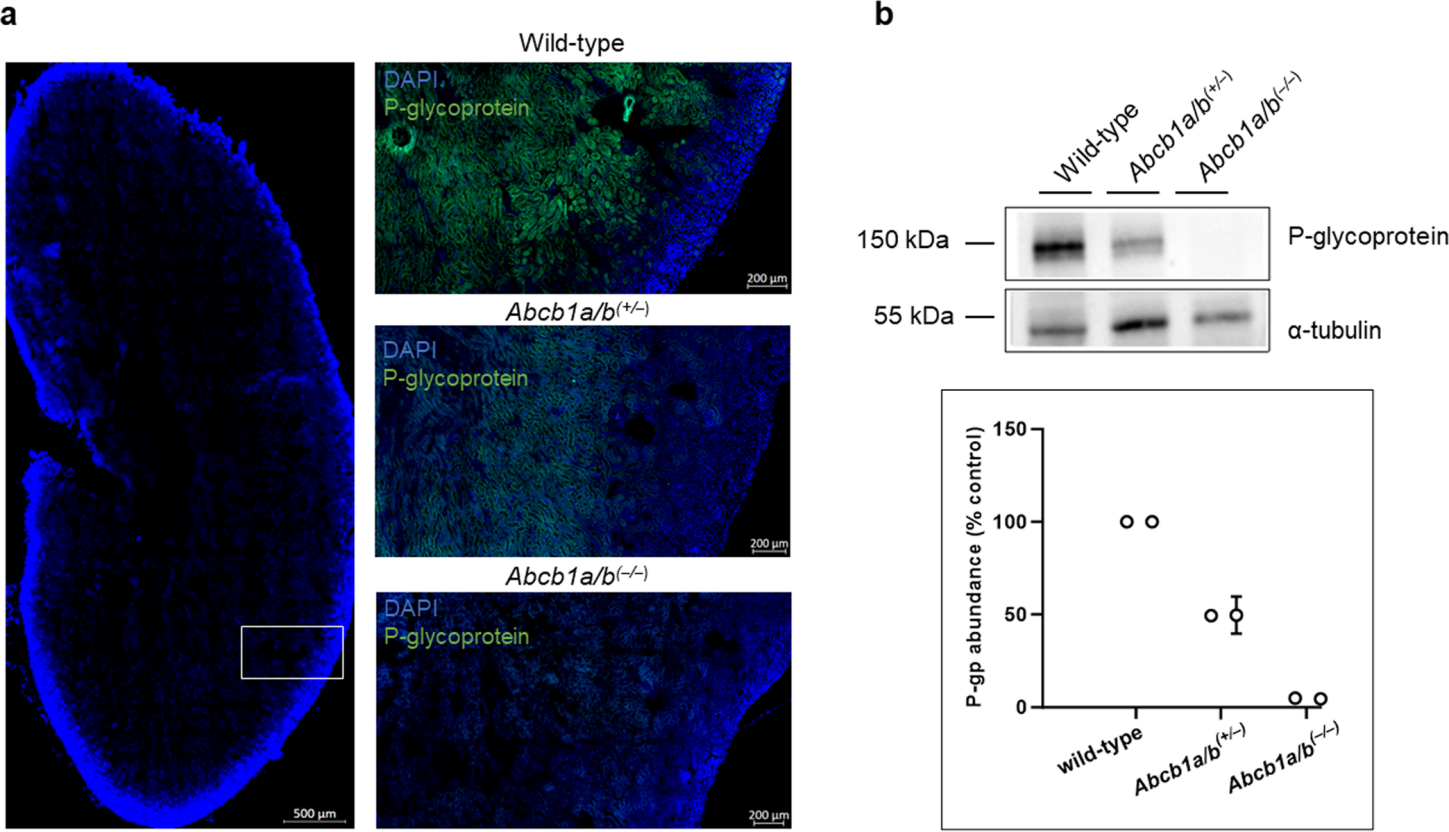
(a) Immunofluorescence labeling of P-gp (green) with counterstained nuclei (DAPI, blue) in kidney sections of wild-type, *Abcb1a/b*^*(+/***–***)*^, and *Abcb1a/b*^*(***–***/***–***)*^ mice. The white rectangle in the left image (scale bar = 500 μm) represents the magnified area shown in the right images (scale bar = 200 μm). (b) Quantification of P-gp abundance in homogenized kidney tissue by Western blotting using α-tubulin as a loading control. Relative P-gp abundance is shown as a percent of control (i.e., wild-type animals). Two animals were analyzed per mouse model with two technical replicates each. Error bars represent the standard deviation.

Immunofluorescence labeling confirmed normal, intermediate, and no P-gp abundance in the kidneys of wild-type, *Abcb1a/b*^*(+/***–***)*^, and *Abcb1a/b*^*(***–***/***–***)*^ mice, respectively (Figure 1a). Western blot analysis revealed a 50% reduction in renal P-gp abundance in *Abcb1a/b*^*(+/***–***)*^ mice and the absence of P-gp in *Abcb1a/b*^*(***–***/***–***)*^ mice as compared with wild-type mice (Figure 1b).

In liver sections of wild-type mice, P-gp could not be successfully visualized with the employed P-gp antibody (see the SI, Figure S2a). Although Western blot analysis indicated presence of P-gp in liver homogenates, no differences in P-gp abundance could be detected between the three mouse models (Figure S2b).

**Figure 2:**
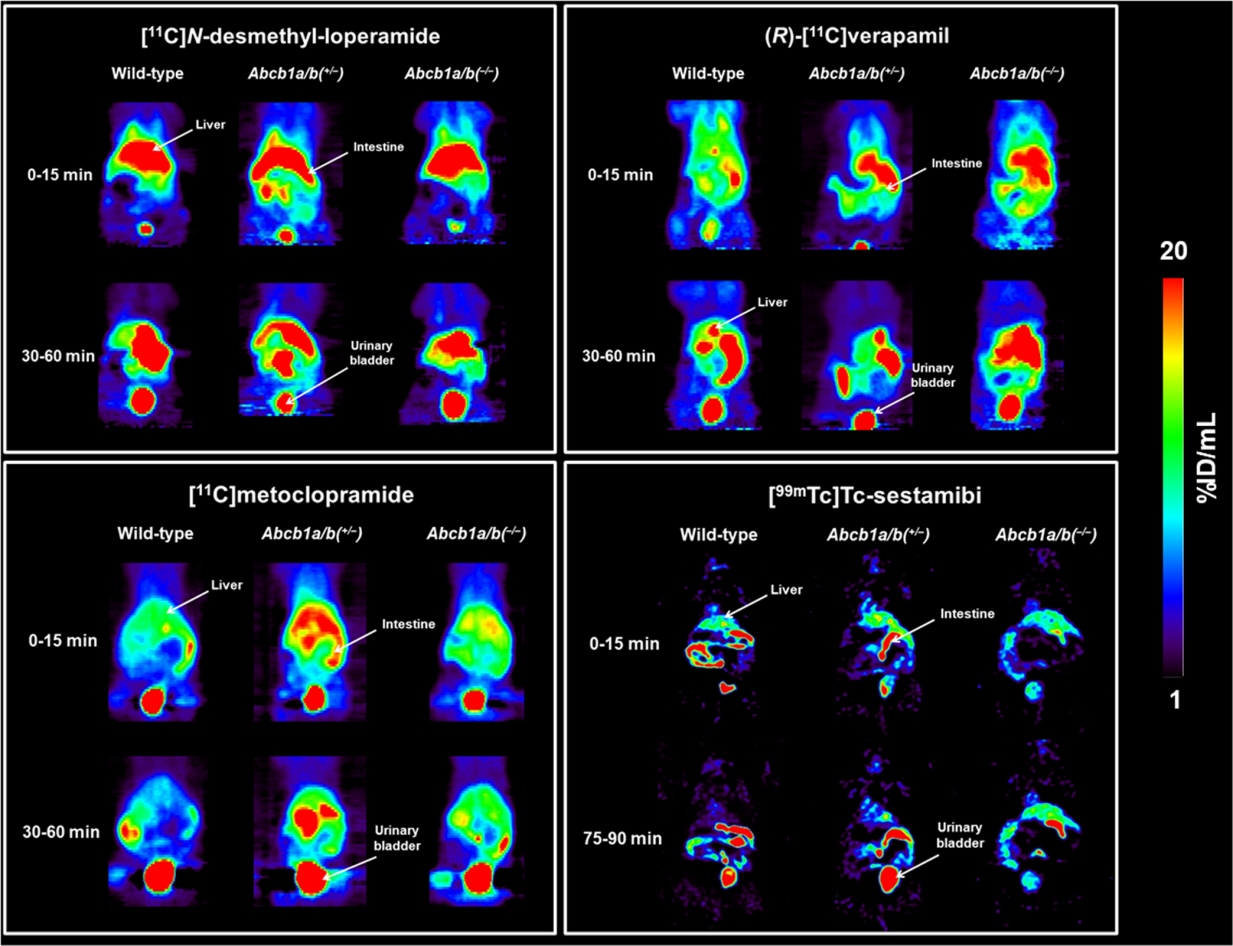
Representative coronal PET summation images and static SPECT images at different time points after i.v. administration of [^11^C]*N*-desmethyl-loperamide, (*R*)-[^11^C]verapamil, [^11^C]metoclopramide, or [^99m^Tc]Tc-sestamibi in wild-type, *Abcb1a/b*^*(+/***–***)*^, and *Abcb1a/b*^*(***–***/***–***)*^ mice. Radioactivity concentration is expressed as the percent of the injected dose per mL (%ID/mL). Anatomical regions are labeled with arrows.

Representative coronal PET and SPECT images of the abdominal region in the early uptake and late elimination phases for all radiotracers in each mouse group are depicted in Figure 2.

**Figure 3:**
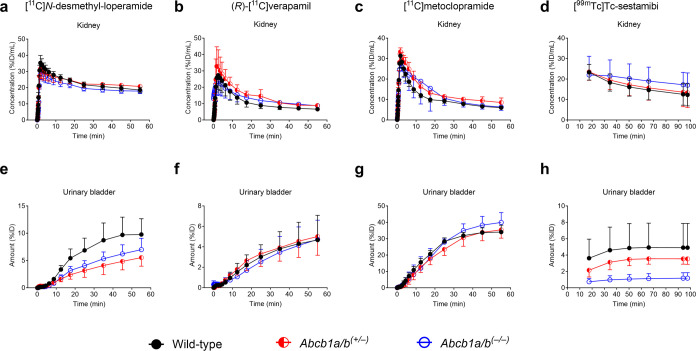
Mean (±SD) time–activity curves (%ID/mL or %ID) in the kidneys (a–d) and urinary bladder (e–h) of wild-type, *Abcb1a/b*^*(+/***–***)*^, and *Abcb1a/b*^*(***–***/***–***)*^ mice after i.v. injection of [^11^C]*N*-desmethyl-loperamide, (*R*)-[^11^C]verapamil, [^11^C]metoclopramide, or [^99m^Tc]Tc-sestamibi.

**Figure 4:**
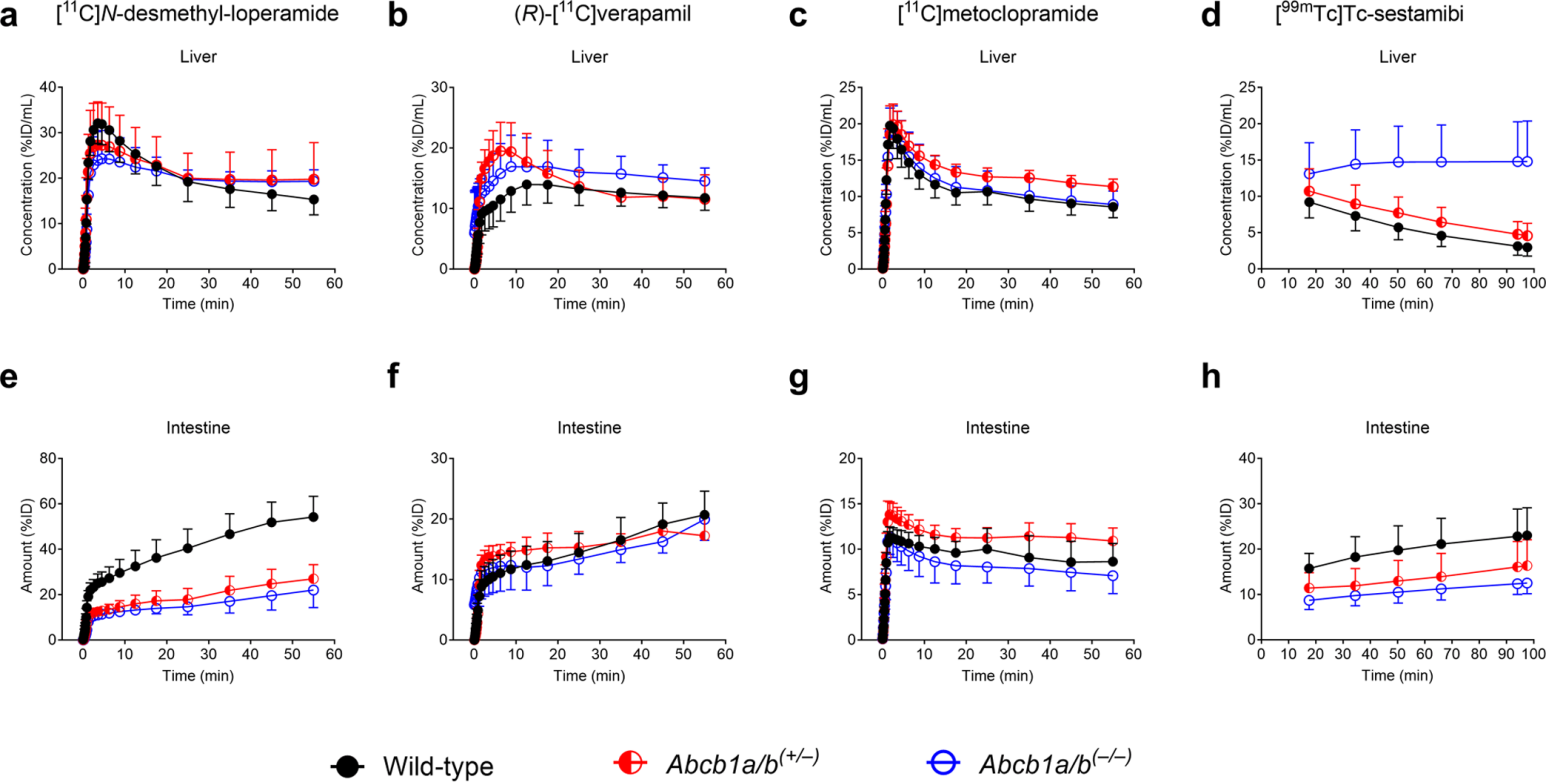
Mean (±SD) time–activity curves (%ID/mL or %ID) in the liver (a–d) and intestine (e–h) of wild-type, *Abcb1a/b*^*(+/***–***)*^, and *Abcb1a/b*^*(***–***/***–***)*^ mice after i.v. injection of [^11^C]*N*-desmethyl-loperamide, (*R*)-[^11^C]verapamil, [^11^C]metoclopramide, or [^99m^Tc]Tc-sestamibi.

For all four radiotracers, the PET and SPECT images revealed radioactivity uptake in the liver as well as the excretion of radioactivity into the intestine and urinary bladder (Figure 2). Mean time–activity curves of all radiotracers in the kidney and urinary bladder are shown in Figure 3 and those in the liver and intestine in Figure 4.

In wild-type animals, most of the radioactivity at 60 min after radiotracer injection was excreted into the bile for [^11^C]*N*-desmethyl-loperamide (54 ± 9%ID), (*R*)-[^11^C]verapamil (21 ± 4%ID), and [^99m^Tc]Tc-sestamibi (22 ± 6%ID), while for [^11^C]metoclopramide, most of the radioactivity was excreted into the urine (34 ± 4%ID).

**Figure 5:**
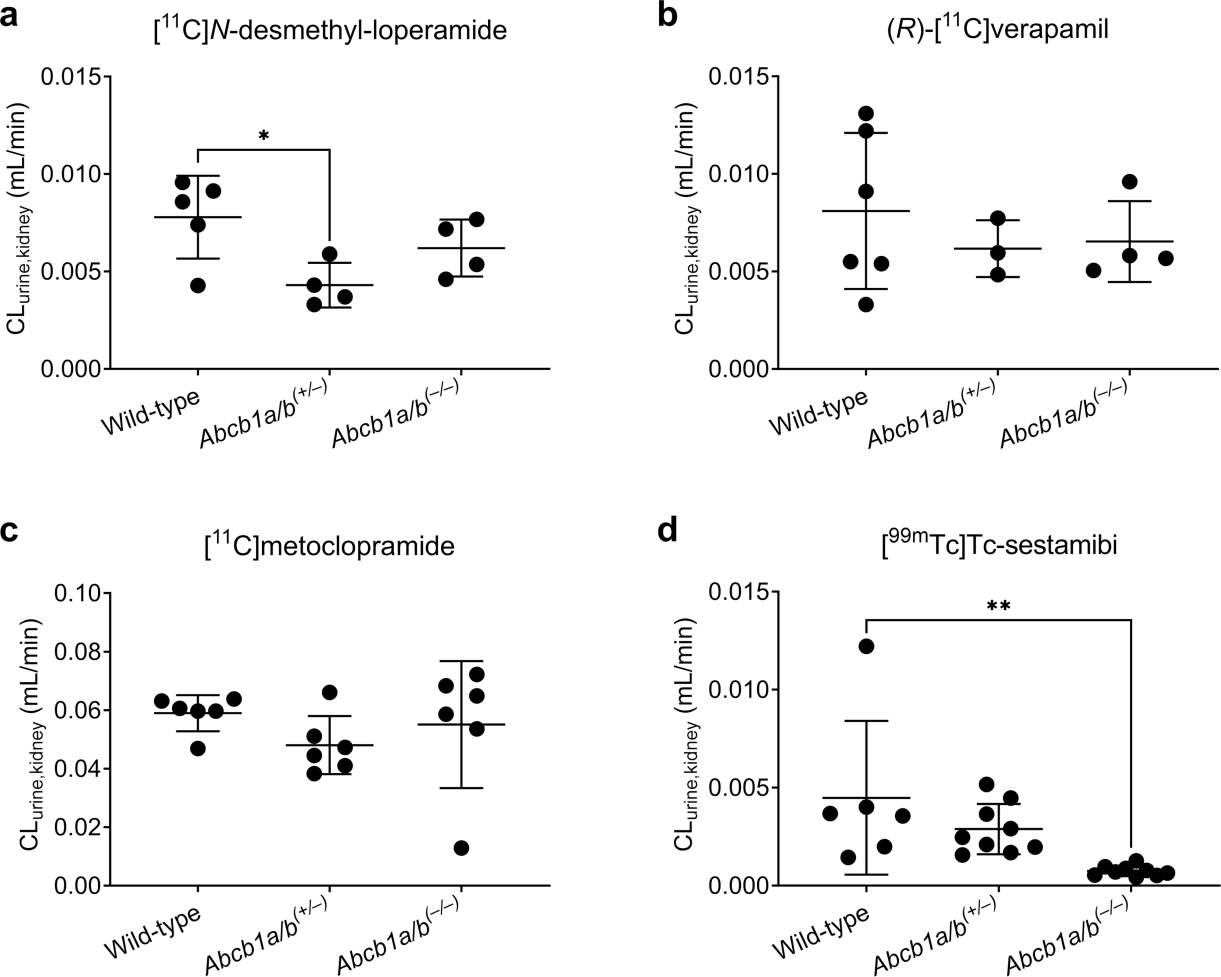
Renal clearance (CL_urine,kidney_) values in wild-type, *Abcb1a/b*^*(+/***–***)*^, and *Abcb1a/b*^*(***–***/***–***)*^ mice after i.v. administration of (a) [^11^C]*N*-desmethyl-loperamide, (b) (*R*)-[^11^C]verapamil, (c) [^11^C]metoclopramide, or (d) [^99m^Tc]Tc-sestamibi. *p ≤ 0.05, **p ≤ 0.01, ordinary one-way ANOVA followed by a Dunnett’s multiple comparison test against the wild-type group.

Renal clearance values (CL_urine,kidney_) in individual animals of all studied mouse groups are shown in Figure 5 for all of the tested radiotracers. Among all radiotracers, CL_urine,kidney_ was significantly lower (−83%) in *Abcb1a/b*^*(***–***/***–***)*^ mice as compared with wild-type mice only for [^99m^Tc]Tc-sestamibi (Figure 5).

In *Abcb1a/b*^*(+/***–***)*^ mice, there was a trend toward a decrease in CL_urine,kidney_ of [^99m^Tc]Tc-sestamibi (−36%), but statistical significance was not reached (Figure 5). Moreover, for [^11^C]*N*-desmethyl-loperamide, CL_urine,kidney_ was significantly lower in *Abcb1a/b*^*(+/***–***)*^ mice as compared with wild-type mice (−45%). No other significant differences were observed in CL_urine,kidney_ between the studied mouse groups for any of the other radiotracers (Figure 5).

**Figure 6:**
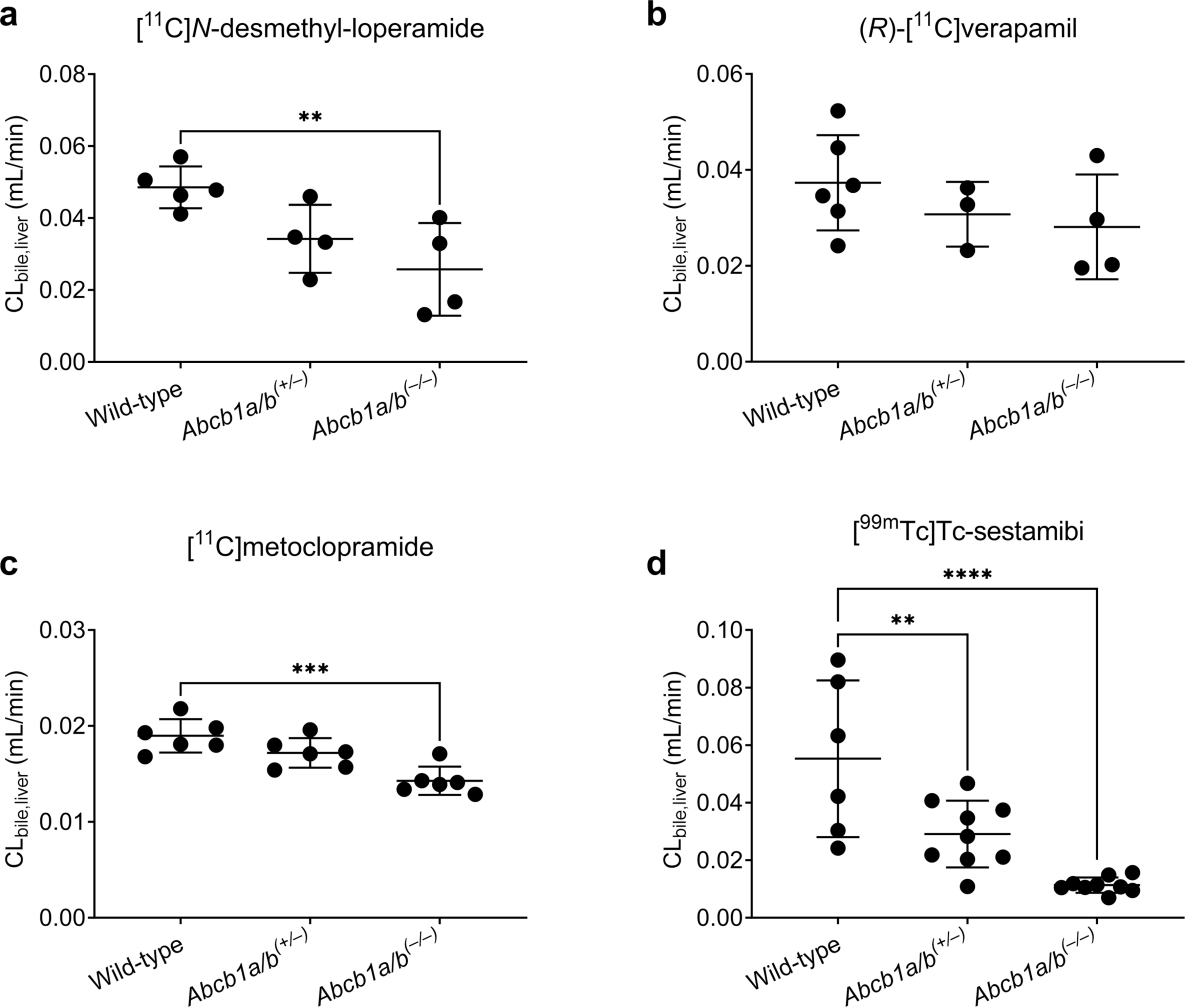
Biliary clearance (CL_bile,liver_) values in wild-type, *Abcb1a/b*^*(+/***–***)*^, and *Abcb1a/b*^*(***–***/***–***)*^ mice after i.v. administration of (a) [^11^C]*N*-desmethyl-loperamide, (b) (*R*)-[^11^C]verapamil, (c) [^11^C]metoclopramide, or (d) [^99m^Tc]Tc-sestamibi. **p ≤ 0.01, ***p ≤ 0.001, ****p ≤ 0.0001, ordinary one-way ANOVA followed by a Dunnett’s multiple comparison test against the wild-type group.

Biliary clearance of radioactivity (CL_bile,liver_) was significantly lower in *Abcb1a/b*^*(***–***/***–***)*^ mice as compared with wild-type mice for [^11^C]*N*-desmethyl-loperamide (−47%), [^11^C]metoclopramide (−25%), and [^99m^Tc]Tc-sestamibi (−79%). In *Abcb1a/b*^*(+/***–***)*^ mice, CL_bile,liver_ was significantly decreased (−47%) only for [^99m^Tc]Tc-sestamibi (Figure 6).

There was a trend toward a decrease in CL_bile,liver_ in *Abcb1a/b*^*(+/***–***)*^ mice as compared with wild-type mice for [^11^C]*N*-desmethyl-loperamide (−30%) and [^11^C]metoclopramide (−9%), but statistical significance was not reached. There was also a trend toward a decrease in CL_bile,liver_ for (*R*)-[^11^C]verapamil in both *Abcb1a/b*^*(+/***–***)*^ (−18%) and *Abcb1a/b*^*(***–***/***–***)*^ mice (−25%) with respect to wild-type mice, but these differences were also not significant (Figure 6).

At the end of the scans, total radioactivity concentrations in venous blood samples were determined for all radiotracers (see the SI, Figure S3). There were no significant differences in blood radioactivity concentrations between mouse groups except for a significantly lower blood concentration for (*R*)-[^11^C]verapamil in *Abcb1a/b*^*(+/***–***)*^ versus wild-type mice.

## Discussion

In this study, we compared the performance and sensitivity of some radiolabeled P-gp substrates for PET with the SPECT radiotracer [^99m^Tc]Tc-sestamibi for measuring P-gp function in the kidneys and liver, using wild-type, *Abcb1a/b*^*(+/***–***)*^, and *Abcb1a/b*^*(***–***/***–***)*^ mice as models of different P-gp abundance in excretory organs. Among the tested radiotracers, [^99m^Tc]Tc-sestamibi performed best in measuring changes in the renal and hepatic P-gp function. Since [^99m^Tc]Tc-sestamibi is commercially available and widely used in clinical nuclear medicine for myocardial perfusion imaging,^26^ it may present an attractive tool for functional imaging of P-gp in excretory organs.

We have recently proposed heterozygous *Abcb1a/b* knockout mice as a model to assess the sensitivity of P-gp substrate radiotracers to measure moderate decreases in P-gp function at the BBB.^17^ Using the same mouse models that were used in the present study, we could show that [^11^C]metoclopramide has a better sensitivity than [^11^C]*N*-desmethyl-loperamide and (*R*)-[^11^C]verapamil to detect an approximately 30% reduction in cerebral P-gp abundance as it occurs in *Abcb1a/b*^*(+/***–***)*^ mice relative to wild-type mice.^17^ To examine the suitability of these mouse models for measuring changes in renal and hepatic P-gp function, we performed immunofluorescence labeling and Western blot analysis of P-gp in the kidneys and liver. These experiments revealed a 50% decrease in the abundance of P-gp in the kidneys of *Abcb1a/b*^*(+/***–***)*^ mice relative to wild-type mice, while no P-gp was detected in *Abcb1a/b*^*(***–***/***–***)*^ mice (Figure 1). This is in good agreement with the differences in cerebral P-gp abundance observed in the same mouse models.^17,23^ Regrettably, the employed anti-P-gp antibody proved to be unsuitable for detecting P-gp in the liver (Figure S2), so hepatic P-gp abundance could not be quantified in the employed mouse models.

An important consideration for the use of PET and SPECT in the assessment of renal and hepatic P-gp function is the inability of these imaging methods to distinguish the radiolabeled parent drug from radiolabeled metabolites. There is a considerable substrate/inhibitor overlap between P-gp and CYP3A enzymes, i.e., substrates/inhibitors of P-gp are often also substrates/inhibitors of CYP3A enzymes.^27^ If a radiolabeled P-gp probe substrate that is metabolized by CYP3A enzymes is used in an imaging-based DDI assessment, changes in P-gp function cannot necessarily be distinguished from changes in CYP3A enzyme activity caused by the perpetrator drug based on the measurement of total radioactivity concentrations in tissue. Therefore, ideally, an effective PET/SPECT probe for measurement of the systemic P-gp function should not be metabolized at all during the time course of the PET/SPECT scan. If metabolism occurs, it should (i) at least not involve CYP3A enzymes and (ii) radiolabeled metabolites should show a minor contribution to the imaging signal in the investigated organ. [^11^C]metoclopramide is mainly metabolized by CYP2D6^28,29^ and chronic treatment of rats with the CYP3A4 and CYP2B6 inducer carbamazepine had no effect on *in vivo* [^11^C]metoclopramide metabolism.^30^ [^11^C]*N*-desmethyl-loperamide has been designed as a metabolically stable PET probe in humans,^31^ whose metabolism in mice was only affected to a minor extent by the potent CYP3A4 inhibitor ketoconazole.^32^ On the other hand, (*R*)-[^11^C]verapamil is extensively metabolized involving CYP3A enzymes.^33−35^ [^99m^Tc]Tc-sestamibi was found to be hardly metabolized *in vivo* in guinea pigs.^36^

Another important consideration is the selectivity of the employed radiotracer for P-gp over other apical ABC efflux transporters expressed in kidney proximal tubule cells or hepatocytes, such as breast cancer resistance protein (BCRP, encoded in humans by the *ABCG2* gene and in rodents by the *Abcg2* gene) or multidrug resistance-associated protein 2 (MRP2, encoded in humans by the *ABCC2* gene and in rodents by the *Abcc2* gene). All four investigated radiotracers are not transported by BCRP.^37−40^ Besides being a P-gp substrate, [^99m^Tc]Tc-sestamibi is a substrate of multidrug resistance-associated protein 1 (MRP1, encoded by the *ABCC1/Abcc1* genes),^37,41^ but this transporter is not expressed in the apical membranes of kidney proximal tubule cells or hepatocytes. After i.v. injection of [^99m^Tc]Tc-sestamibi, biliary recovery of radioactivity and liver radioactivity profiles measured with planar imaging were similar in MRP2-deficient TR^***–***^ rats and in wild-type rats, suggesting that MRP2 does not play a role in the biliary excretion of [^99m^Tc]Tc-sestamibi.^42^

Compartmental pharmacokinetic models have proven useful to analyze changes in drug disposition caused by alterations in membrane transporter abundance and function.^25,43−46^ Compartmental models are based on physiologically based pharmacokinetic models, in which the compartments correspond to predefined organs or tissues interconnected by blood flow. In addition, the input of the system in PET kinetic modeling is typically a directly measured arterial blood or plasma curve. However, for PET or SPECT imaging in small animals such as mice, it is challenging to perform dynamic blood sampling due to the small blood volume of mice and the inaccessibility of animals once positioned in the gantry of the scanner. The generation of an image-derived blood input function (i.e., from the left ventricle of the heart)^45^ was also not feasible for all investigated radiotracers, as (*R*)-[^11^C]verapamil and [^99m^Tc]Tc-sestamibi had high myocardial uptake potentially resulting in contamination of the image-derived blood signal. Therefore, due to the lack of a reliable blood input function for the investigated radiotracers and in order to consistently analyze the obtained PET and SPECT data in a comparable manner, and thanks to the rich dynamic data that is provided by imaging methods, we performed a semicompartmental analysis by calculating the renal and biliary clearances with respect to the kidney and liver concentrations (CL_urine,kidney_ and CL_bile,liver_), respectively. This allowed us to quantitatively assess changes in P-gp function at the apical membranes of both renal proximal tubule cells and hepatocytes in the different studied mouse models.

CL_urine,kidney_ of [^99m^Tc]Tc-sestamibi was reduced by 36% in *Abcb1a/b*^*(**+**/***–***)*^ mice and by 83% in *Abcb1a/b*^*(***–***/***–***)*^ mice as compared with wild-type mice (Figure 5), which is in good agreement with the reductions in protein abundance found with immunofluorescence labeling and Western blot analysis (50 and 100% reduction in *Abcb1a/b*^*(+/***–***)*^ and *Abcb1a/b*^*(***–***/***–***)*^ mice, respectively) (Figure 1). This suggests that [^99m^Tc]Tc-sestamibi is a sensitive radiotracer to measure changes in the P-gp function in kidney proximal tubule cells. While [^99m^Tc]Tc-sestamibi has been used before to assess P-gp function in the liver of mice,^47^ this is, to our knowledge, the first time that this radiotracer is used to measure renal P-gp function. For the investigated PET radiotracers, no significant differences in CL_urine,kidney_ were observed among mouse groups (except for [^11^C]*N*-desmethyl-loperamide in *Abcb1a/b*^*(****+****/***–***)*^ mice) (Figure 5), suggesting a lack of suitability of these radiotracers to measure renal P-gp function.

In agreement with the results obtained for renal clearance and in accordance with earlier literature results,^47^ CL_bile,liver_ of [^99m^Tc]Tc-sestamibi was significantly reduced (−79%) in *Abcb1a/b*^*(***–***/***–***)*^ mice (Figure 6). Further than that, CL_bile,liver_ of [^99m^Tc]Tc-sestamibi was significantly reduced by 47% in *Abcb1a/b*^*(****+****/***–***)*^ mice (Figure 6), which suggested that this radiotracer has good sensitivity to measure moderate changes in P-gp function in hepatocytes, as they may occur in clinical DDIs when hepatic P-gp is only partially inhibited. However, these findings could not be related to differences in protein abundance in the liver tissue of the mouse models, as the employed P-gp antibody was not suitable to quantify hepatic P-gp. The apparently higher sensitivity of [^99m^Tc]Tc-sestamibi to measure hepatic as compared with renal P-gp function may be explained by the higher absolute values of CL_bile,liver_ (Figure 6) as compared with CL_urine,kidney_ (Figure 5). In addition to the changes in CL_bile,liver_ of [^99m^Tc]Tc-sestamibi, CL_bile,liver_ of [^11^C]*N*-desmethyl-loperamide was also significantly reduced (−47%) in *Abcb1a/b*^*(***–***/***–***)*^ mice, but not in *Abcb1a/b*^*(+/***–***)*^ mice (Figure 6). Therefore, although [^11^C]*N*-desmethyl-loperamide may show some suitability to measure hepatic P-gp function, it is not as sensitive as [^99m^Tc]Tc-sestamibi, which may be due to the formation of non-P-gp transported radiolabeled metabolites of [^11^C]*N*-desmethyl-loperamide in mice. This might also explain the lack of sensitivity of (*R*)-[^11^C]verapamil and [^11^C]metoclopramide to detect changes in renal and hepatic P-gp function. Previous studies have shown that at 25 min after i.v. radiotracer injection, only 13 and 47% of total radioactivity in mouse plasma were derived from the parent radiotracer for [^11^C]*N*-desmethyl-loperamide and (*R*)-[^11^C]verapamil,^22^ respectively, and only 39% of total radioactivity in mouse plasma represented the parent radiotracer at 15 min after [^11^C]metoclopramide injection.^21^ At the same time point, 78 and 66% of total radioactivity in the liver and kidney, respectively, represented parent [^11^C]metoclopramide.^21^ Therefore, it appears possible that changes in P-gp-mediated renal or hepatic clearance of [^11^C]*N*-desmethyl-loperamide, (*R*)-[^11^C]verapamil, and [^11^C]metoclopramide were masked by the presence of radiolabeled metabolites that are not transported by P-gp. However, in humans, metabolism of [^11^C]metoclopramide and [^11^C]*N*-desmethyl-loperamide is considerably slower than in mice,^7,31^ which raises the possibility that these radiotracers may possess better sensitivity to measure changes in P-gp function in the human liver.

Apart from its good metabolic stability, another possible explanation for the higher sensitivity of [^99m^Tc]Tc-sestamibi compared with the PET tracers to measure renal and hepatic P-gp function could be a lower passive permeability of [^99m^Tc]Tc-sestamibi, which is a permanent cation. This could result in a higher proportion of P-gp-mediated transport relative to passive diffusion at the apical membranes of kidney proximal tubule cells and hepatic cells.

In comparison to PET, SPECT is more widely used in clinical nuclear medicine. While the synthesis of ^11^C-labeled PET radiotracers requires the availability of an onsite medical cyclotron, SPECT radiotracers such as [^99m^Tc]Tc-sestamibi can be straightforwardly prepared using commercially available kits and a ^99m^Tc-generator. Compared with PET, SPECT has a lower sensitivity for detection of radioactivity. This led in our study to the necessity for longer imaging time frames (15 min) and consequently a loss of temporal resolution for [^99m^Tc]Tc-sestamibi kinetics as compared with the PET radiotracers. However, our results showed that for the analysis of renal and biliary excretion, the temporal solution attainable with the employed SPECT system was sufficient to obtain sensitive measures of renal and hepatic P-gp function with [^99m^Tc]Tc-sestamibi. For a future translation of the [^99m^Tc]Tc-sestamibi SPECT imaging protocol to humans, it needs to be considered that unlike the employed preclinical SPECT camera, the transaxial field of view (FOV) of clinical SPECT systems (approximately 40 cm) will not cover all excretory organs. Therefore, in humans, the urinary bladder will be outside the FOV of clinical SPECT scanners. However, the amount of radioactivity excreted into the urine can be straightforwardly determined by gamma counter measurements of urine collected at the end of the SPECT scan. A clinical SPECT protocol that allows for the measurement of renal and hepatic P-gp function may find application in clinical drug development to assess transporter-mediated DDIs or to assess the effect of disease on transporter function.

## Conclusions

We compared the performance and sensitivity of [^11^C]*N*-desmethyl-loperamide, (*R*)-[^11^C]verapamil, and [^11^C]metoclopramide with [^99m^Tc]Tc-sestamibi for measuring P-gp function in the kidneys and liver of mice. Among the tested radiotracers, [^99m^Tc]Tc-sestamibi performed best in measuring renal and hepatic P-gp function. Given its commercial availability, its widespread clinical use, and its good metabolic stability, [^99m^Tc]Tc-sestamibi appears as an attractive probe substrate to assess P-gp-mediated DDIs at the level of excretory organs and to measure P-gp function under different (patho-)physiological conditions. Furthermore, our data support the idea that heterozygous *Abcb1a/b* knockout mice are a suitable model to assess the sensitivity of P-gp radiotracers to measure moderate changes in systemic P-gp function.

## Supporting Information Available

PET images illustrating the delineation of different regions of interest (Figure S1); immunofluorescence labeling and Western blot analysis of P-gp in the liver of wild-type, *Abcb1a/b^(+/−)^*, and *Abcb1a/b^(−/−)^* mice (Figure S2); venous blood concentrations in wild-type, *Abcb1a/b^(+/−)^*, and *Abcb1a/b^(−/−)^* mice at the end of the scan for all radiotracers (Figure S3) (PDF)
