# Supplementary material for: Performance and Sensitivity of [99mTc]Tc-sestamibi Compared with Positron Emission Tomography Radiotracers to Measure P-glycoprotein Function in the Kidneys and Liver
Source: Mol Pharm. 2024 Jan 16;21(2):932–43. doi: 10.1021/acs.molpharmaceut.3c01036 (PMC10848257; doi:10.1021/acs.molpharmaceut.3c01036)
Supplement: Supplementary file 1 — mp3c01036_si_001.pdf [file mp3c01036_si_001.pdf]

Supporting Information

Performance and sensitivity of [^{99m}Tc]Tc-sestamibi compared with PET radiotracers to measure P-glycoprotein function in the kidneys and liver

Irene Hernández-Lozano[1], Sarah Leterrier[2], Severin Mairinger[1,3], Johann Stanek[3], Anna S. Zacher[3], Lara Breyer[3], Marcus Hacker[3], Markus Zeitlinger[1], Jens Pahnke[4,5,6,7], Nicolas Tournier[2], Thomas Wanek[3], Oliver Langer[1,3,*]

[1] Department of Clinical Pharmacology, Medical University of Vienna, 1090 Vienna, Austria

[2] Laboratoire d'Imagerie Biomédicale Multimodale (BIOMAPS), Université Paris-Saclay, CEA, CNRS, Inserm, Service Hospitalier Frédéric Joliot, 91401 Orsay, France

[3] Department of Biomedical Imaging and Image-guided Therapy, Medical University of Vienna, 1090 Vienna, Austria

[4] Department of Pathology, Section of Neuropathology, Translational Neurodegeneration Research and Neuropathology Lab, University of Oslo (UiO) and Oslo University Hospital (OUS), 0372 Oslo, Norway

[5] Lübeck Institute of Experimental Dermatology (LIED), Pahnke Lab, University of Lübeck and University Medical Center Schleswig-Holstein, 23538 Lübeck, Germany

[6] Department of Pharmacology, Faculty of Medicine, University of Latvia, 1004 Rīga, Latvia

[7] Department of Neurobiology, The Georg S. Wise Faculty of Life Sciences, Tel Aviv University, 6997801 Tel Aviv, Israel

Corresponding author: Oliver Langer, Department of Clinical Pharmacology, Medical University of Vienna, A-1090 Vienna, Austria. Tel.: +43 1 40400-29810, Fax: +43 1 40400-29980, e-mail: oliver.langer@meduniwien.ac.at

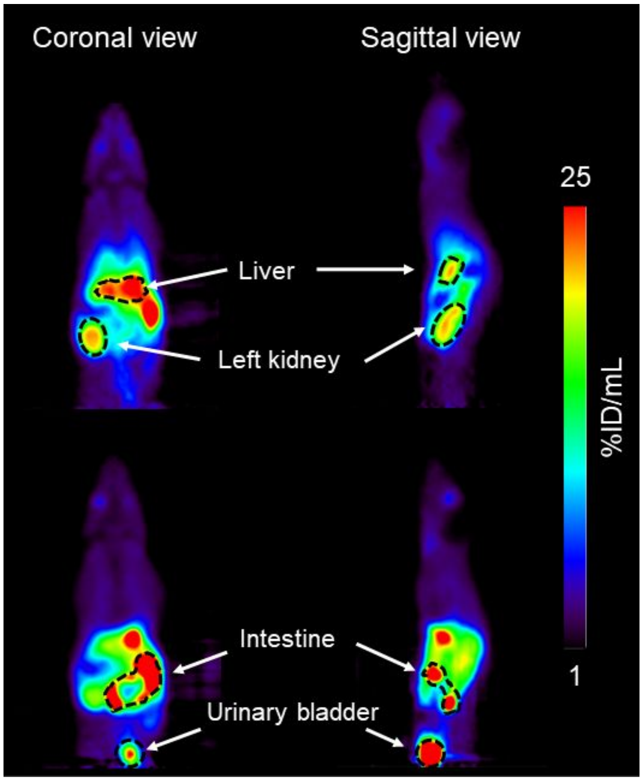

Figure S1. Delineation of regions of interest. Shown are representative coronal and sagittal PET summation images (0-15 minutes for the upper images and 15-60 minutes for the lower images) of a wild-type mouse injected with [^{11}C]*N*-desmethyl-loperamide. Regions of interest are outlined with black dashed lines and labeled with arrows.

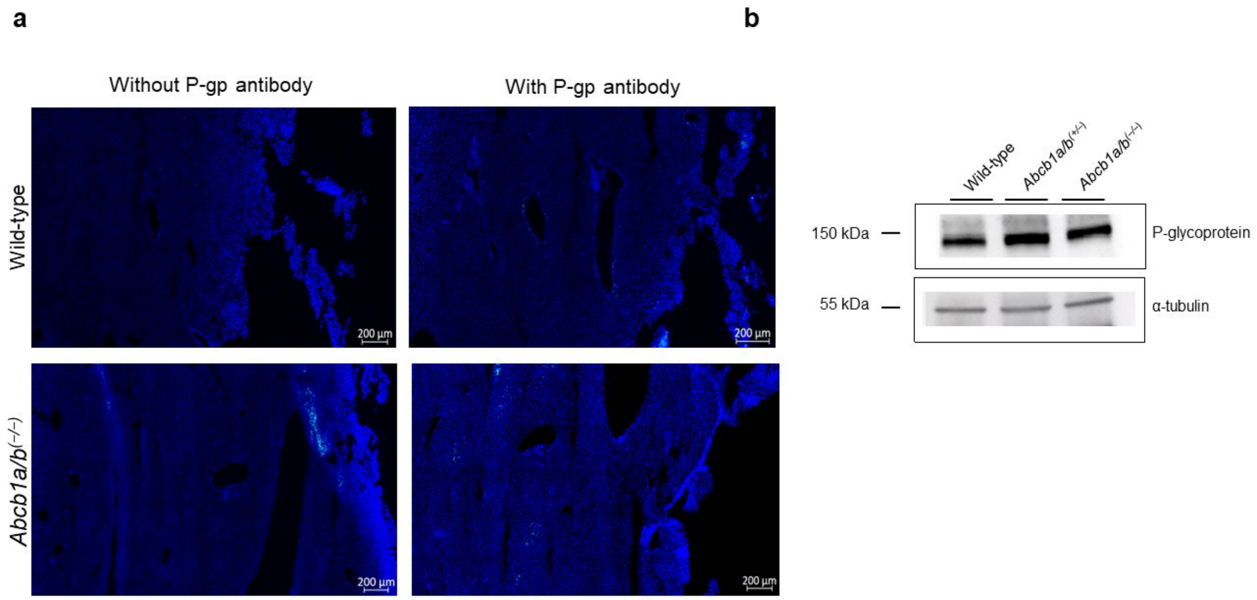

Figure S2. (a) Immunofluorescence labeling of P-gp (green, not visible) with counterstained nuclei (DAPI, blue) in liver sections of wild-type mice (upper row) and *Abcb1a/b*$^{(–/–)}$ mice (lower row) (scale bars = 200 µm). Both immunofluorescence labeling without (left column, used as control) and with (right column) the P-gp antibody is depicted. (b) Analysis of P-gp in homogenized liver tissue by Western blotting using α-tubulin as a loading control.

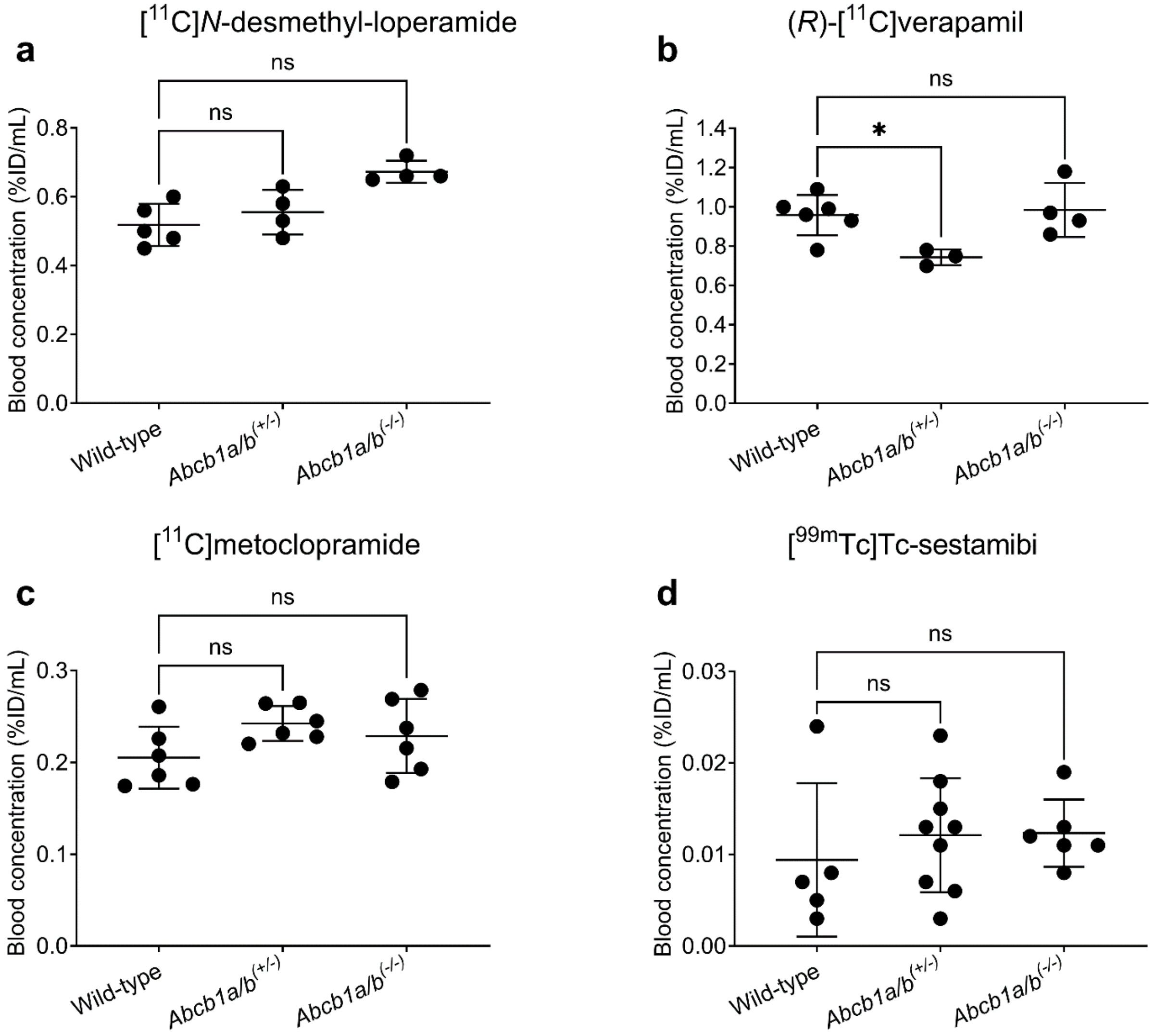

Figure S3. Venous blood concentrations (%ID/mL) in wild-type, *Abcb1a/b*$^{(+/–)}$ and *Abcb1a/b*$^{(–/–)}$ mice at the end of the scan for (a) [^{11}C]*N*-desmethyl-loperamide, (b) (*R*)-[^{11}C]verapamil, (c) [^{11}C]metoclopramide and (d) [^{99m}Tc]Tc-sestamibi. ns, not significant, *$p \leq 0.05$, ordinary one-way ANOVA followed by a Dunnett's multiple comparison test against the wild-type group.
